# Associations between non-traditional lipid parameters and normoglycemic reversion in Chinese adults with prediabetes: a retrospective analysis

**DOI:** 10.3389/fendo.2025.1502861

**Published:** 2025-06-24

**Authors:** Cheng Huang, Yuhang Zhang, Xiaoqing Yang, Guofeng Li, Zhichao Gao

**Affiliations:** ^1^ Department of Colorectal Surgery, First People’s Hospital of Xiaoshan District, Hangzhou, Zhejiang, China; ^2^ Department of Orthopedics, First People’s Hospital of Xiaoshan District, Hangzhou, Zhejiang, China; ^3^ Department of Endocrinology, First People’s Hospital of Xiaoshan District, Hangzhou, Zhejiang, China; ^4^ Department of Neurosurgery, First People’s Hospital of Xiaoshan District, Hangzhou, Zhejiang, China

**Keywords:** prediabetes, normoglycemia, lipid parameters, chinese, atherogenic index of plasma (AIP)

## Abstract

**Background:**

Prediabetes is a critical precursor to type 2 diabetes and poses an increasing global health challenge, particularly in China. While many individuals with prediabetes can revert to normoglycemia through lifestyle interventions, the impact of non-traditional lipid parameters on this process requires further investigation. Understanding the associations between these lipid parameters and glycemic recovery could inform more effective prevention strategies.

**Methods:**

This study analyzed data from the Dryad public database, focusing on a cohort of 14,735 Chinese adults with prediabetes. Multivariate logistic regression models assessed the association between twelve lipid parameters and normoglycemia. Two-piecewise logistic regression was used to identify inflection points. Stratified and sensitivity analyses were conducted by age, sex, BMI, and family history of diabetes. The Area Under the Curve was calculated to compare predictive performance.

**Results:**

During a mean follow-up of 2.94 years, 6,406 out of 14,735 participants with prediabetes (56.53%) reverted to normoglycemia. Multivariate logistic regression showed that lower levels of the atherogenic index of plasma (AIP), remnant cholesterol (RC), RC/HDL-C ratio, and LDL-C, as well as higher HDL-C, were significantly associated with a higher likelihood of normoglycemia. Except for HDL-C, all lipid parameters showed non-linear relationships with normoglycemia. While all lipid indices displayed modest discriminative ability (AUC > 0.5), AIP demonstrated the highest area under the curve (AUC = 0.579, 95% CI: 0.569–0.588). Subgroup and sensitivity analyses confirmed stronger associations in females, younger individuals (< 50 years), and those with lower BMI (< 24 kg/m²).

**Conclusion:**

AIP, RC, and the RC/HDL-C ratio were strongly associated with reversion to normoglycemia among Chinese adults with prediabetes. AIP showed the strongest and most consistent relationship, especially in younger females with lower BMI.

## 1 Introduction

Prediabetes, characterized by intermediate hyperglycemia, serves as a critical precursor to diabetes and cardiovascular complications (1). Globally, the prevalence of prediabetes among adults reached 5.8% in 2021 and is projected to rise to 6.5% by 2045 (2). In China, this condition presents an even greater public health challenge: while the national diabetes prevalence stands at 12.8%, a staggering 35.2% of the population is classified as prediabetic (3). This metabolic state is highly dynamic. Up to 37% of untreated prediabetes cases may progress to type 2 diabetes within four years, but early intervention to restore normoglycemia can substantially reduce cardiovascular risks (4, 5). Restoring blood glucose levels to normal can significantly reduce the future incidence of chronic cardiovascular disease (6). However, there is limited research on similar topics. This reversibility highlights the urgent need to identify modifiable predictors of glycemic recovery.

Dyslipidemia frequently coexists with prediabetes, and elevated low-density lipoprotein cholesterol (LDL-C) and triglycerides (TG) being well-documented risk factors (7, 8). Currently, non-traditional lipid parameters—including the atherogenic index of plasma (AIP), non-HDL-C/HDL-C ratio, and total cholesterol (TC)/HDL-C ratio—may provide superior predictive value for dysglycemia progression (9–13). However, their specific roles in predicting the reversal of prediabetes remain underexplored. This is a clinically vital distinction given the divergent management goals between disease prevention and regression. Notably, recent studies indicate that reductions in remnant cholesterol (RC) and non-HDL-C/HDL-C ratio correlate with prediabetes regression to normoglycemia,implying these parameters may serve as both biomarkers and therapeutic targets (14, 15). Only a few studies have examined the restoration of normoglycemia in pre-diabetic patients, and there has been no systematic evaluation of unconventional lipid parameters in this context. As a result, it remains unclear which parameters are the best predictors.

We hypothesize that specific non-traditional lipid parameters have varying predictive capacities for normoglycemia restoration in prediabetes Chinese adults. In contrast to previous studies, the present research aims to identify the best predictors by systematically assessing the role of 12 lipid parameters in reversing prediabetes.

## 2 Methods

### 2.1 Data sources and study population

**Figure 1:**
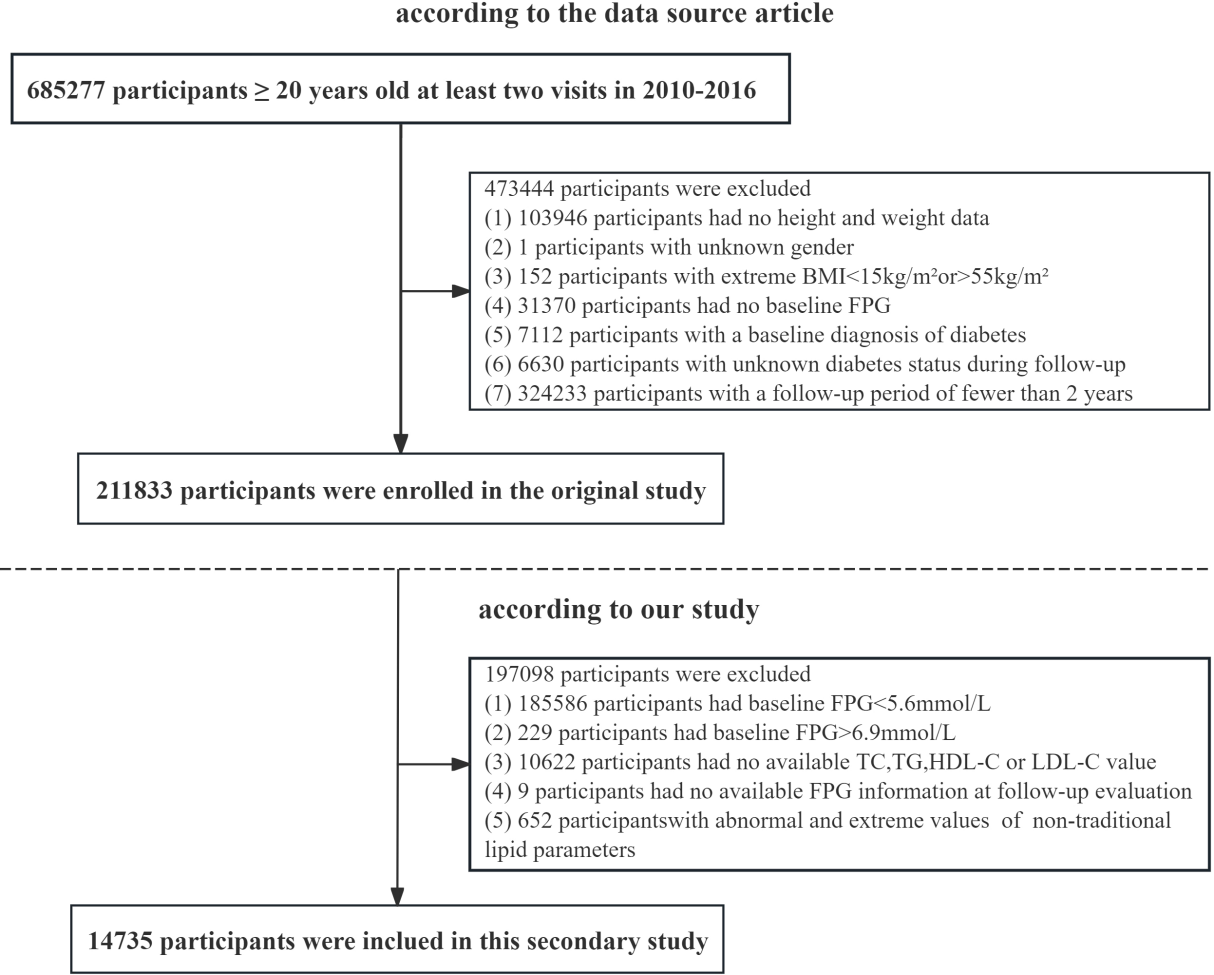
Flow chart of study participants.

This study is a retrospective analysis. This study obtained approval from the Rich Healthcare Group Review Board, and the data were collected retrospectively. The data has been uploaded to the open-access database Dryad (16). This dataset recorded the health examination information of personnel from the Rich Healthcare Group from 2010 to 2016. The dataset includes 211,833 individuals and was gathered from 2010 to 2016 across numerous regions in China. At baseline, the original study excluded participants for the following reasons: 103,946 individuals lacked available measurements for weight and height; 1 participant had no information on gender; 152 individuals presented extreme body mass index (BMI) values (either < 15 kg/m² or > 55 kg/m²); and 31,370 participants did not have fasting plasma glucose values available. Additionally, we excluded participants who had visit intervals shorter than two years (n = 324,233) and those diagnosed with diabetes, which included 2,997 individuals identified through self-report and 4,115 individuals with a fasting plasma glucose level of ≥ 7.0 mmol/L. The researchers also excluded participants with an undefined diabetes status at follow-up (n = 6,630). Ultimately, a total of 211,833 participants were included in the original analysis. According to the research objectives, we performed a secondary selection of the population. Participants with fasting plasma glucose (FPG) levels outside the range of 5.6–6.9 mmol/L (n = 185,815) were excluded from the study. Those with missing data on HDL-C, total cholesterol (TC), LDL-C, TG, or FPG were also excluded (n = 10,631). In addition, participants whose values for any non-traditional lipid parameters exceeded three standard deviations from the mean were omitted (n = 652). Ultimately, 14,735 participants were included in the final analysis (8,329 males and 6,406 females) (**Figure 1**).

### 2.2 Data collection

At each visit, participants underwent a structured health evaluation that included questionnaire-based and physical measurement components. The baseline assessment included a comprehensive questionnaire designed to elicit detailed information about personal medical history, participants’ lifestyle habits, demographic characteristics, and family history of diabetes. Physical measurements taken during each visit included blood pressure, height and weight. Additionally, information on smoking and alcohol consumption habits was collected at the beginning of the study. Participants were classified into one of three categories for each habit: current user, former user, or never user. Blood samples were obtained from each participant after they had fasted for a minimum of 10 hours. Blood samples were subsequently analyzed for a range of biochemical parameters, including HDL-C, FPG, TC, TG, LDL-C, and additional metabolic markers. Biochemical analyses were performed using a Beckman 5800 autoanalyzer, ensuring consistency and reliability of the results (16).

RC/HDL-C ratio = RC/HDL-C (17);
Non-HDL-C = TC − HDL-C (18).
Castelli’s index-II (CRI-II) is defined as the ratio of LDL-C to HDL-C (19);
Castelli’s index-I (CRI-I) is calculated as the ratio of TC to HDL-C (19);
Atherogenic coefficient (AC) is calculated as the ratio of non-HDL-C to HDL-C (10);
Lipoprotein combine index (LCI) = (TC × TG × LDL-C)/HDL-C (20);
AIP = lg (TG/HDL-C) (13);
remnant cholesterol (RC)  = TC − HDL-C − LDL-C (21);

### 2.3 Definitions

According to the 2018 American Diabetes Association criteria, participants with FPG levels from 5.6 to 6.9 mmol/L are classified as having prediabetes (22). During the follow-up period, participants with FPG levels below 5.6 mmol/L who did not self-report a diagnosis of diabetes were categorized into the Normoglycemia group. Individuals whose FPG exceeds 5.6 mmol/L during the follow-up period will be categorized into the hyperglycemia group. Dyslipidemia was identified by any of the following criteria: TG ≥ 150 mg/dL, TC ≥ 200 mg/dL, HDL-C < 50 mg/dL in women or < 40 mg/dL in men, or LDL-C ≥ 130 mg/dL. Additionally, participants who were receiving lipid-lowering therapy were considered to have dyslipidemia (23).

### 2.4 Statistical analysis

Categorical variables were depicted using frequency counts and percentages. Continuous variables were expressed as mean (standard deviation) or median (interquartile range). The ANOVA and Kruskal-Wallis tests were utilized to evaluate the differences in continuous variables across various groups, while the chi-square test was employed to assess categorical variables.To address concerns regarding collinearity among the covariates, we calculated the variance inflation factor (VIF) in the multivariate models (24). Variables with a VIF exceeding a certain threshold (> 5) were deemed to exhibit collinearity. The VIF of all variables included in this study is less than 5 (**Supplementary Table S1**). Multiple imputation methods were employed to handle missing data, aiming to minimize the variability introduced by the absence of certain variables (25). Multivariate logistic regression model analysis the likelihood of reverting to normoglycemia in participants with prediabetes. Model 1 was calculated without adjusting for covariates. Model 2 further adjusted age, sex, SBP, DBP, BMI, family history of diabetes, and lifestyle habits (smoking and alcohol status). Model 3 further adjusted FPG, aminotransferase (ALT and AST), blood urea nitrogen (BUN), and serum creatinine (Scr) based on model 2. The variables adjusted in the multivariate logistic regression model were guided by clinical knowledge and previously published research (15, 26, 27). After adjusting for all covariates, All lipid parameters were converted into quartiles. Furthermore, a generalized additive model was used to examine the dose-response relationship between lipid parameters and normoglycemia, as well as any potential inflection points. Subsequently, we plotted Receiver Operating Characteristic (ROC) curves for each subgroup. In order to evaluate the predictive performance of each non-traditional lipid parameter, we further calculated the Area Under the Curve (AUC) to evaluate the predictive performance of each non-traditional lipid parameter. Stratified and sensitivity analyses were conducted based on age, sex, BMI, and family history of diabetes. To validate the robustness of the primary outcomes across different populations, individuals with dyslipidemia were excluded. Analyses were performed using EmpowerStats software. A two-tailed P value of less than 0.05 was deemed statistically significant.

## 3 Results

### 3.1 Baseline characteristics of participants

The final cohort comprised 14,735 prediabetic Chinese adults (mean age 50.91 ± 13.51 years; 64.3% male). Within an average follow-up period of 2.94 years, 6,406 participants (56.53%) reverted to normoglycemia. Compared to those who remained hyperglycemic, individuals achieving normoglycemia were significantly younger (47.49 ± 13.39 vs. 53.54 ± 13.01 years, P < 0.001) and more likely to be female (39.92% vs. 32.45%, P < 0.001). They also exhibited lower BMI (24.16 ± 3.27 vs. 25.25 ± 3.27 kg/m²), SBP (124.11 ± 16.99 vs. 129.99 ± 17.78 mmHg), and DBP (76.68 ± 10.87 vs. 79.75 ± 11.24 mmHg; all P < 0.001). All traditional and non-traditional lipid parameters (including TC, TG, LDL-C, LCI, AIP, RC, and RC/HDL-C ratio) were significantly lower in the normoglycemic group (P < 0.001), while HDL-C levels were higher (1.37 ± 0.29 vs. 1.33 ± 0.28 mmol/L, P < 0.001) (**Table 1**).

**Table 1:** Baseline characteristics of participants.

Characteristic	Overall	Hyperglycemia	Normoglycemia	*P* value
Participants	14735	8329	6406	
Age, years	50.91 ± 13.51	53.54 ± 13.01	47.49 ± 13.39	< 0.001
Sex				< 0.001
Female, n (%)	9475 (64.30%)	2703 (32.45%)	2557 (39.92%)	
Male, n (%)	5260 (64.30%)	5626 (67.55%)	3849 (60.08%)	
BMI, kg/m^2^	24.78 ± 3.32	25.25 ± 3.27	24.16 ± 3.27	< 0.001
SBP, mmHg	127.43 ± 17.68	129.99 ± 17.78	124.11 ± 16.99	< 0.001
DBP, mmHg	78.41 ± 11.18	79.75 ± 11.24	76.68 ± 10.87	< 0.001
FPG, mmol/L	5.95 ± 0.32	6.04 ± 0.34	5.84 ± 0.24	< 0.001
TC, mmol/L	4.98 ± 0.89	5.02 ± 0.89	4.93 ± 0.88	< 0.001
TG, mmol/L	1.67 ± 1.04	1.76 ± 1.05	1.54 ± 1.00	< 0.001
HDL-C, mmol/L	1.35 ± 0.29	1.33 ± 0.28	1.37 ± 0.29	< 0.001
LDL-C, mmol/L	2.91 ± 0.67	2.92 ± 0.66	2.88 ± 0.67	< 0.001
ALT, U/L	27.71 ± 22.84	29.20 ± 24.99	25.76 ± 19.52	< 0.001
AST, U/L	26.01 ± 11.54	26.72 ± 12.38	25.10 ± 10.27	< 0.001
BUN, mmol/L	5.00 ± 1.24	5.05 ± 1.24	4.94 ± 1.25	< 0.001
Scr, μmol/L	72.98 ± 16.16	73.73 ± 15.99	72.01 ± 16.33	< 0.001
LCI	20.08 ± 15.82	21.52 ± 16.15	18.20 ± 15.18	< 0.001
AIP	0.03 ± 0.28	0.06 ± 0.28	-0.01 ± 0.28	< 0.001
non-HDL-C	3.64 ± 0.83	3.69 ± 0.83	3.56 ± 0.83	< 0.001
AC	2.82 ± 0.90	2.89 ± 0.90	2.73 ± 0.89	< 0.001
CRI-I	3.82 ± 0.90	3.89 ± 0.90	3.73 ± 0.89	< 0.001
CRI-II	2.23 ± 0.63	2.27 ± 0.62	2.18 ± 0.64	< 0.001
RC	0.73 ± 0.42	0.77 ± 0.42	0.68 ± 0.41	< 0.001
RC/HDL-C ratio	0.59 ± 0.40	0.62 ± 0.41	0.55 ± 0.38	< 0.001
Smoking status, n (%)				< 0.001
Current	3760 (25.52%)	2239 (26.88%)	1521 (23.74%)	
Once	661 (4.49%)	368 (4.42%)	293 (4.57%)	
Never	10314 (70.00%)	5722 (68.70%)	4592 (71.68%)	
drinking status, n (%)				0.105
Current	664 (4.51%)	401 (4.81%)	263 (4.11%)	
Once	2772 (18.81%)	1574 (18.90%)	1198 (18.70%)	
Never	11299 (76.68%)	6354 (76.29%)	4945 (77.19%)	
family histroy of diabetes, n (%)	382 (2.59%)	217 (2.61%)	165 (2.58%)	0.911

Values are n(%), mean ± SD or medians (quartiles).

TC total cholesterol, TG triglyceride, HDL-C high-density lipoprotein cholesterol, LDL-C low-density lipoprotein cholesterol, LCI lipoprotein combine index, AIP atherogenic index of plasma, AC atherogenic coefficient, CRI-I Castelli’s index-I, CRI-II Castelli’s index-II, RC remnant cholesterol.

### 3.2 Relationship between lipid parameters and normoglycemia

**Figure 2:**
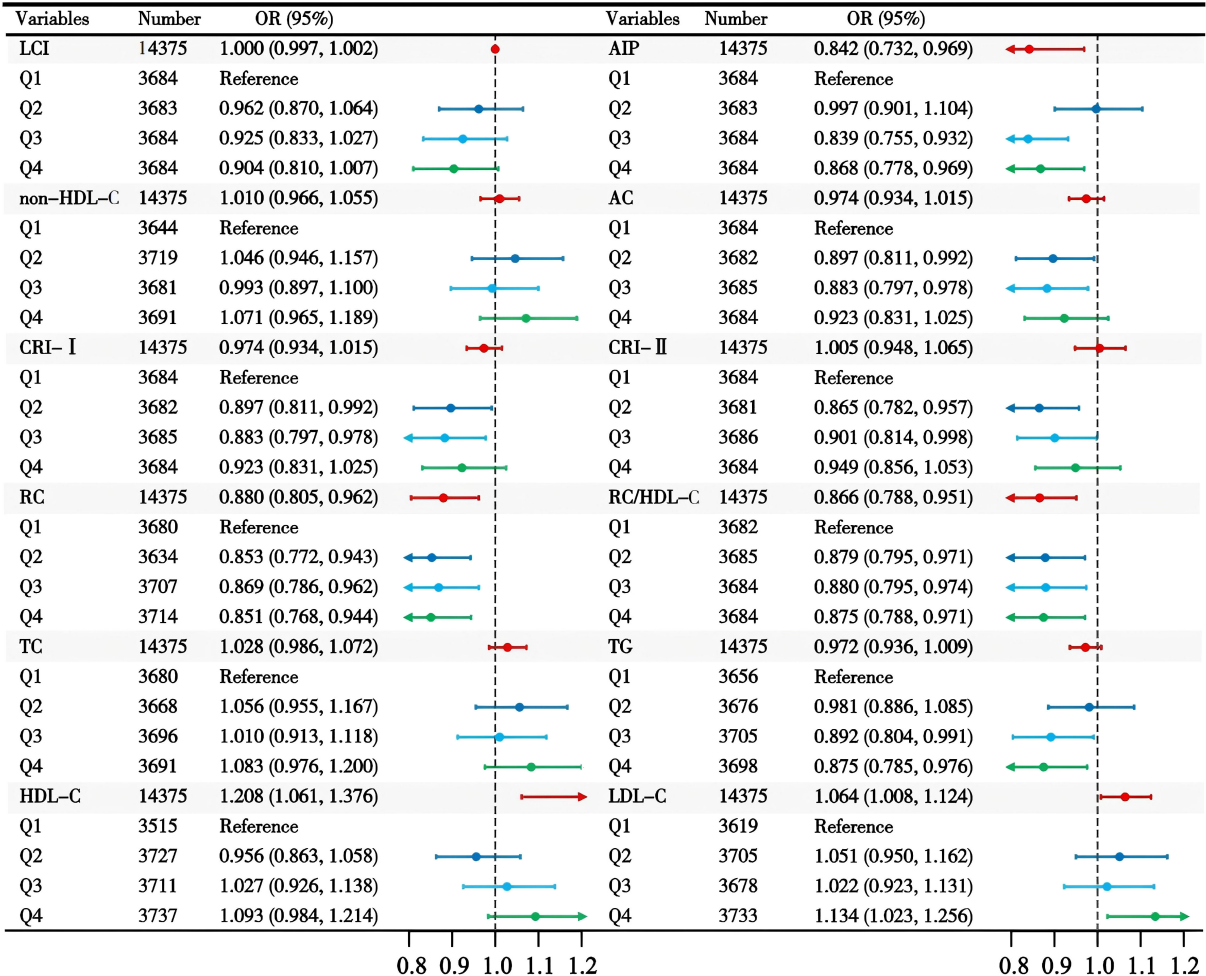
The association between lipid parameters and reversion to normoglycemia in individuals with prediabetes.

Multivariate logistic regression identified AIP, RC, RC/HDL-C ratio, HDL-C, and LDL-C as significant predictors of normoglycemia after adjusting for covariates (**Table 2**). In Model 1 (unadjusted), all lipid parameters were significantly associated with normoglycemia. However, after adjustment for demographic characteristics, family history of diabetes, and lifestyle habits in Model 2, the associations for CRI-II and non-HDL-C became non-significant after adjustment (*P* > 0.05). In Model 3, which adjusted for all variables, AIP, RC, the RC/HDL-C ratio, HDL-C, and LDL-C maintained significant associations with normoglycemia. Specifically, an increase in AIP by one unit was associated with reductions of 61.0%, 30.7%, and 15.8% in the odds of reverting to normoglycemia in Models 1, 2, and 3, respectively. Higher levels of RC decreased the odds by 12.0% (OR 0.880, 95% CI: 0.805–0.962), while the RC/HDL-C ratio was associated with a 13.4% lower odds (OR 0.866, 95% CI: 0.788–0.951). Conversely, HDL-C saw nearly doubled odds with each unit increase (OR 1.915, 95% CI: 1.759–2.085), and LDL-C paradoxically increased the odds (OR 1.056, 95% CI: 1.017–1.097). Notably, the associations for CRI-II and non-HDL-C became non-significant after adjusting for demographic and clinical factors (P > 0.05). **Figure 2** illustrates that both RC and the RC/HDL-C ratio consistently displayed negative associations with glucose recovery across quartiles. Furthermore, elevated AIP related to a decreased probability of achieving normoglycemia, particularly at moderate to high levels (*P* < 0.05).

**Table 2:** Multivariate logistic regression analyses of the relationship between lipid parameters and normoglycemia.

Lipid parameters	Model 1	Model 2	Model 3
	OR (95% CI)	OR (95% CI)	OR (95% CI)
LCI	0.986 (0.984, 0.988)	0.996 (0.994, 0.998)	1.000 (0.997, 1.002)
AIP	0.390 (0.347, 0.439)	0.693 (0.607, 0.791)	0.842 (0.732, 0.969)
Non-HDL-C	0.829 (0.797, 0.862)	0.980 (0.939, 1.022)	1.010 (0.966, 1.055)
AC	0.813 (0.784, 0.844)	0.947 (0.910, 0.985)	0.974 (0.934, 1.015)
CRI-I	0.813 (0.784, 0.844)	0.947 (0.910, 0.985)	0.974 (0.934, 1.015)
CRI-II	0.806 (0.765, 0.850)	0.978 (0.925, 1.034)	1.005 (0.948, 1.065)
RC	0.609 (0.562, 0.660)	0.818 (0.751, 0.891)	0.880 (0.805, 0.962)
RC/HDL-C ratio	0.600 (0.551, 0.652)	0.804 (0.735, 0.879)	0.866 (0.788, 0.951)
TC	0.889 (0.865, 0.915)	0.980 (0.953, 1.009)	0.989 (0.961, 1.018)
TG	0.864 (0.841, 0.888)	0.963 (0.936, 0.990)	0.997 (0.970, 1.025)
HDL-C	1.974 (1.827, 2.134)	1.944 (1.787, 2.116)	1.915 (1.759, 2.085)
LDL-C	0.944 (0.910, 0.980)	1.054 (1.015, 1.095)	1.056 (1.017, 1.097)

OR, Odds ratio; CI, confidence interval; LCI lipoprotein combine index, AIP atherogenic index of plasma, AC atherogenic coefficient, CRI-I Castelli’s index-I, CRI-II Castelli’s index-II, RC remnant cholesterol.

Model 1: no covariates were adjusted; Model 2: gender, age, BMI, SBP, DBP, family history of diabetes, smoking and drinking status were adjusted; Model 3: gender, age, BMI, SBP, DBP, family history of diabetes, smoking, drinking status, FPG, ALT, AST, Scr and BUN were adjusted.

### 3.3 Inflection points and nonlinear trends of lipid parameters

**Figure 3:**
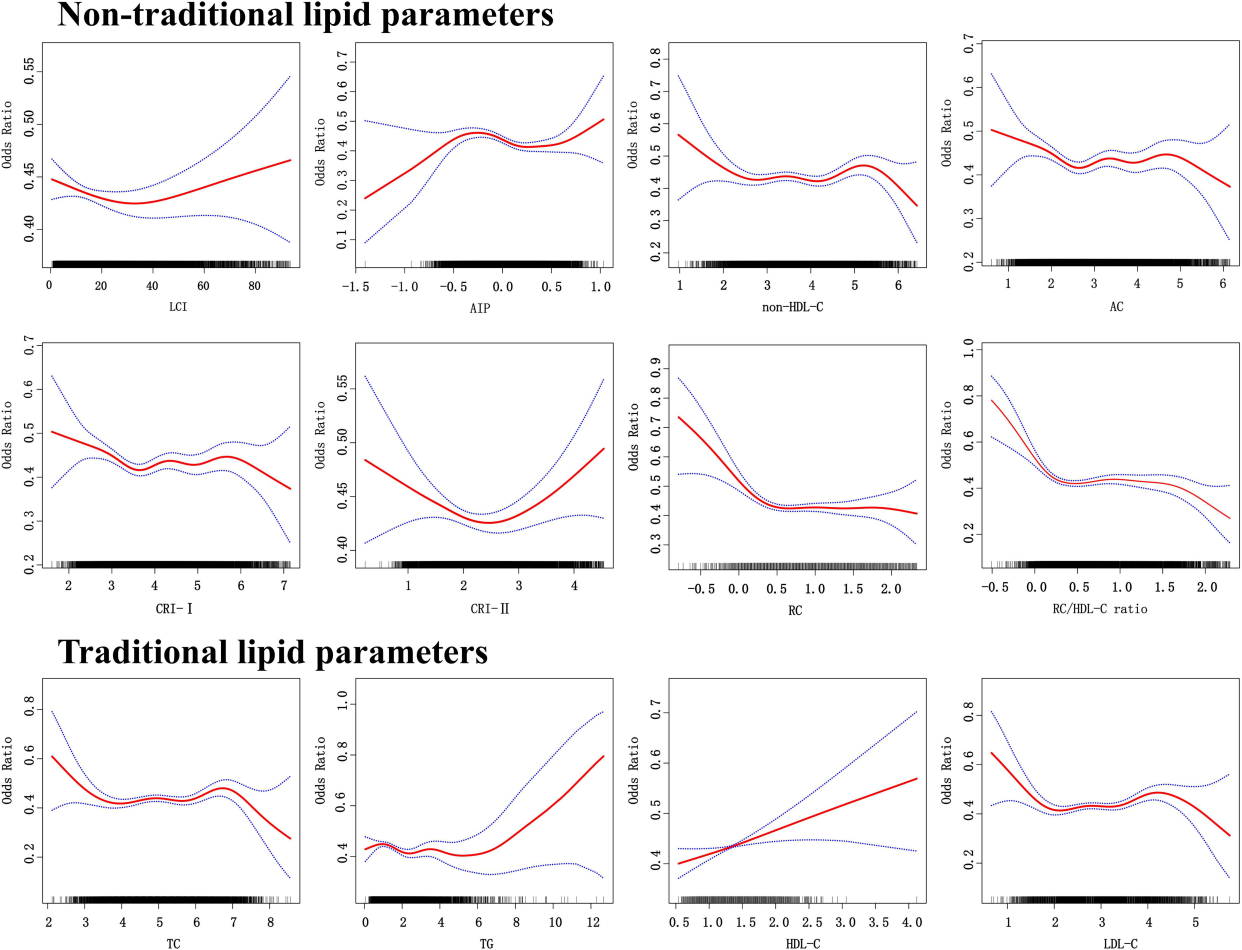
The dose response relationship between lipid parameters and reversion to normoglycemia in people with prediabetes.

The dose-response relationships depicted in **Figure 3** highlight the nonlinear associations between lipid parameters and normoglycemia. Except for HDL-C, all lipid parameters exhibited nonlinear trends (**Supplementary Table S2**). For AIP, a sharp increase in normoglycemia probability occurred below an inflection point of -0.375 (OR 3.034, 95% CI: 1.111–8.286), while above this threshold, the negative association weakened (OR 0.778, 95% CI: 0.667–0.906). Similarly, the RC/HDL-C ratio showed accelerated negative effects below 0.106. above this level, the rate of decrease decelerates.

### 3.4 Diagnostic utility of lipid parameters in normoglycemia identification

**Figure 4:**
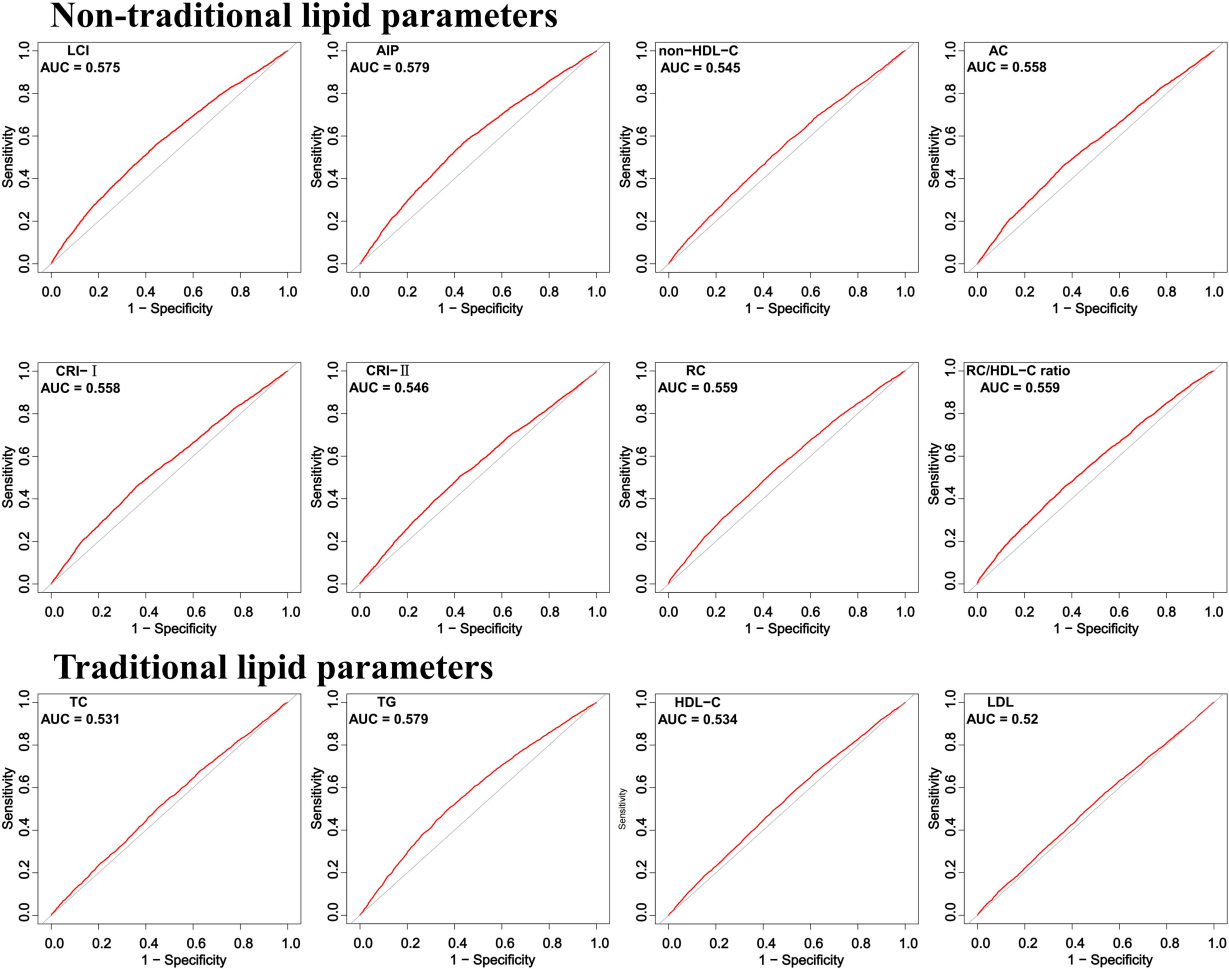
ROC curve analysis of the lipid parameters in predicting normoglycemia.

ROC curve analysis demonstrated that all non-traditional lipid parameters outperformed traditional measures (TG, LDL-C) in predicting normoglycemia reversion (AUC > 0.5). AIP showed the highest discriminative ability (AUC = 0.579, 95% CI: 0.569–0.588), with an optimal cutoff of 0.030 (sensitivity 57.2%, specificity 56.0%). Other strong predictors included LCI (AUC 0.575) and RC (AUC 0.559), while traditional LDL-C performed poorest (AUC 0.520) (**Table 3**; **Figure 4**).

**Table 3:** Ability of traditional and non-traditional lipid parameters to predict normoglycemia in prediabetes.

Variables	AUC	95% CI low	95% CI up	Best threshold	Specificity	Sensitivity
AIP	0.579	0.569	0.588	0.030	0.560	0.572
LCI	0.575	0.566	0.584	14.333	0.584	0.533
non-HDL-C	0.545	0.536	0.555	3.645	0.498	0.575
AC	0.558	0.548	0.567	2.451	0.639	0.459
CRI-I	0.558	0.548	0.567	3.451	0.639	0.459
CRI-II	0.546	0.537	0.556	2.070	0.577	0.507
RC	0.559	0.550	0.568	0.660	0.558	0.529
RC/HDL-C	0.559	0.549	0.568	0.422	0.632	0.456
TC	0.531	0.522	0.540	4.895	0.546	0.507
TG	0.579	0.569	0.588	1.295	0.621	0.506
HDL-C	0.534	0.524	0.543	1.285	0.458	0.595
LDL-C	0.520	0.510	0.529	2.955	0.462	0.574

TC total cholesterol, TG triglyceride, HDL-C high-density lipoprotein cholesterol, LDL-C low-density lipoprotein cholesterol, LCI lipoprotein combine index, AIP atherogenic index of plasma, AC atherogenic coefficient, CRI-I Castelli’s index-I, CRI-II Castelli’s index-II, RC remnant cholesterol, AUC area under the receiver operating characteristic curve, CI confdence interval.

### 3.5 Sensitivity analyses

**Figure 5:**
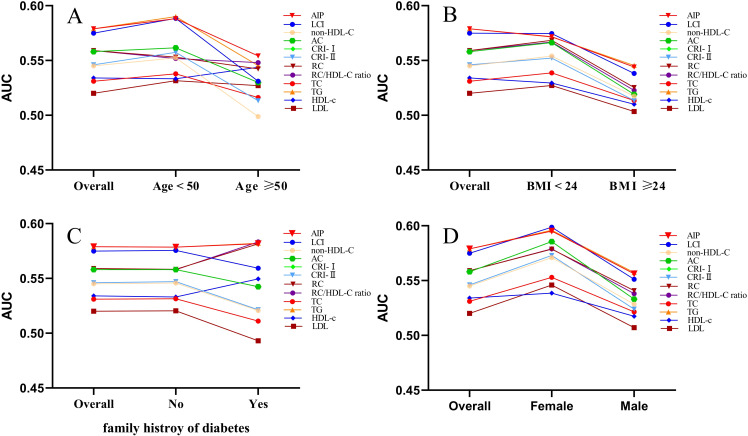
The AUC of lipid parameters in stratified analysis by age **(A)**, BMI **(B)**, family history of diabetes **(C)** and sex **(D)**.

Stratification by key covariates revealed stronger associations in specific subgroups (**Figure 5**). In terms of sex, all lipid parameters showed stronger effects in females compared to males. Age-related associations were more pronounced in participants under 50 years, while effects were heightened in individuals with a BMI less than 24 kg/m². Additionally, stronger correlations were observed in those without a familial risk for diabetes. After excluding participants with baseline dyslipidemia, the AIP consistently emerged as the most robust marker for normoglycemia, with the lowest OR value after adjusting for all variables (model 3) (OR 0.708, 95% CI: 0.551–0.910), and AIP demonstrated the strongest predictive ability (**Supplementary Tables S3**, **S4**). Importantly, the relationship between AIP and normoglycemia remained significant regardless of age, weight, BMI, or diabetes family history, reinforcing the consistency of our primary findings (**Supplementary Table S5**).

## 4 Discussion

This study systematically evaluates the associations and diagnostic significance of twelve lipid parameters in relation to normoglycemia among prediabetic Chinese adults. In this study, 6,406 individuals (56.53%) successfully returned to normoglycemia within an average observation period of 2.94 years. Among the parameters analyzed, only AIP, RC, RC/HDL-C ratio, HDL-C,and LDL-C showed significant association with normoglycemia. Notably, AIP demonstrated the highest diagnostic efficacy for predicting reversion to normoglycemia. Moreover, younger females with lower BMI were more likely to rrestore normal blood glucose levels.

The capacity for individuals with prediabetes to regain glucose control varies notably amaong different ethnic groups. For instance, a longitudinal study involving 9,637 Mexican individuals revealed that only 22.6% reverted to normoglycemia within a 2.5 year period (28). In contrast, 54% of participants in the UK with prediabetes returned to normoglycemia in just one year (29). Similar research results showed that 44.9% of prediabetic participants in China returned to normoglycemia after a mean follow-up period of 8.75 years (6). Furthermore, the incidence of cardiovascular events in those who returned to normoglycemia was significantly lower compared to those who developed diabetes. These findings emphasize the importance of actively managing prediabetes. Our study’s finding that 56.53% of prediabetic individuals successfully regained normoglycemia within 2.94 years highlights the potential effectiveness of targeted intervention strategies for reversing prediabetes. In comparison to select populations such as those of Chinese descent, the Mexican population exhibits a higher incidence and greater management challenges for prediabetes and diabetes, which can be attributed to a multifactorial pathogenesis. Specifically, the synergistic interaction of hypercaloric, carbohydrate-rich dietary patterns, sedentary lifestyle, and genetic susceptibility creates a permissive environment for disease development (30, 31). Socioeconomic disparities further exacerbate this landscape, as low-income demographics demonstrate disproportionate dependence on cost-effective, calorie-dense processed foods. Additionally, sociocultural factors and systemic healthcare disparities pose significant barriers to effective glycemic management, thereby perpetuating the epidemic (32, 33).

Elevated levels of lipids accelerate the progression of prediabetes, highlighting the importance of managing dyslipidemia to improve blood glucose control (3, 34, 35). Non-traditional lipid parameters, which encompass lipid indicators linked to cardiovascular health, extend beyond conventional markers (12). Historically, research has concentrated on the role of non-traditional lipid parameters in cerebrovascular diseases, including ischemic stroke and asymptomatic intracranial arterial stenosis (36, 37). Recent findings have confirmed that abnormal non-traditional lipid parameters elevate the risk of developing prediabetes (38, 39). Additionally, there is a negative nonlinear association between RC and the probability of Chinese prediabetic adults achieving normal blood glucose levels (15). Despite the established associations, limited attention has been given to the impact of non-traditional lipid parameters on achieving normoglycemia in individuals with prediabetes (15). In our study, after adjusting for all covariates, we found that AIP, RC, and the RC/HDL-C ratio were significant associations with normoglycemia. AIP is a composite indicator derived from TG and HDL-C. HDL-C is known to facilitate reverse cholesterol transport and exert regulatory effects on inflammation (40). The antioxidant and anti-inflammatory functions of HDL-C are impaired in individuals with diabetes (41). Of these parameters, AIP had the highest diagnostic utility in predicting the return to normoglycemia. This finding is particularly important as AIP is recognized as a critical modifiable risk factor for cardiovascular events (42). Furthermore, if AIP levels are not effectively managed, the risk of developing diabetes may increase (13, 43). Specifically, maintaining an AIP value below -0.375 significantly improves the chances of individuals with prediabetes reverting to normoglycemia.

The mechanisms by which lipid profiles influence normoglycemia in prediabetic patients require further exploration. The prevalence of diabetes is rapidly increasing among Asian populations, with new cases emerging at relatively younger ages and lower body mass indices compared to Western populations. This trend may be related to poorer pancreatic β-cell reserves (44). While the absence of functional β-cells is a critical factor in diabetes, their maintenance and renewal depend on self-replication (45). The capacity for β-cell self-replication declines with age, indicating that younger individuals may have a greater potential for β-cell regeneration (46). Elevated cholesterol and other lipid levels can lead to β-cell dysfunction, further complicating glucose metabolism (12, 47). Estrogen can significantly promote insulin secretion, protect the function of pancreatic β-cells, delay their failure, and maintain the stability of insulin secretion (48). The development of prediabetes represents a complex pathophysiological process. While its current diagnostic criteria primarily rely on FPG levels, integrating magnetic resonance imaging-derived metrics of fat distribution, hepatic lipid content, and polygenic risk scores could enhance patient stratification and enable precision therapeutic strategies (49).

Interestingly, our study reveals that younger prediabetic individuals and those with lower BMI are more likely to achieve normoglycemia. Additionally, men are more susceptible to obesity, and hyperglycemia when facing nutritional challenges compared to women (50). Compared to men, young women are more likely to normalize their blood glucose levels, potentially attributable to greater emphasis on health management and higher treatment compliance (51). Smoking and drinking habits are more prevalent among males, while a healthy lifestyle may play a crucial role in controlling and reversing hyperglycemic conditions (52). This contrasts with older populations, who may be more prone to unhealthy dietary habits and a sedentary lifestyle, thereby elevating their risk of developing diabetes (53). While pharmacological interventions can be beneficial, changes in lifestyle, such as adopting a healthier diet and engaging in regular physical activity, are essential for effectively managing prediabetes (54). For individuals with prediabetes, reducing calorie intake along with increasing physical activity can significantly decrease the risk of progressing to diabetes (55). Therefore, such interventions may effectively assist individuals with prediabetes in returning to normal blood glucose levels (56).

This study has several significant strengths. First, it is the first comprehensive assessment of the relationship between lipid parameters and normoglycemia in Chinese adults with prediabetes. Second, the research is based on a large cohort of 14,735 Chinese prediabetic patients, enhancing the reliability and generalizability of the findings. Finally, the study highlights that younger females and individuals with a low BMI are more likely to attain normal blood glucose levels, which helps prioritize key demographics for targeted prevention efforts in managing prediabetes.

This study has several limitations. First, as our participants were exclusively Chinese, the generalizability of the AIP threshold to other ethnicities is uncertain, and further research is needed to explore its applicability in populations with diverse genetic backgrounds. Additionally, we assessed non-traditional lipid parameters only at baseline, without considering their temporal fluctuations over time. Although we discussed possible ethnic variations in glycemic recovery rates, we did not perform comparative analyses of population-specific thresholds for key lipid parameters, such as AIP cutoff values across Chinese, Mexican, and UK cohorts. Moreover, the absence of oral glucose tolerance and HbA1c data in our cohort may have led to an underestimation of prediabetes prevalence. Finally, the lack of baseline information on gestational diabetes mellitus, dyslipidemia, and metabolic syndrome may influence the stability and generalizability of our findings.

## 5 Conclusion

In this study, AIP, RC, and the RC/HDL-C ratio demonstrated stronger associations with normoglycemia in Chinese adults. Notably, AIP exhibited the highest diagnostic efficacy in predicting glycemic recovery. However, future studies are required to replicate these findings externally and validate the generalizability of the AIP threshold across diverse racial and metabolic populations.

## Data Availability

The datasets presented in this study can be found in online repositories. The names of the repository/repositories and accession number(s) can be found below: Dryad public database (https://datadryad.org/stash/dataset/doi:10.5061/dryad.ft8750v).
