# Supplementary material for: Associations between non-traditional lipid parameters and normoglycemic reversion in Chinese adults with prediabetes: a retrospective analysis
Source: Front Endocrinol (Lausanne). 2025 Jun 24;16:1502861. doi: 10.3389/fendo.2025.1502861 (PMC12234296; doi:10.3389/fendo.2025.1502861)
Supplement: Supplementary file 1 [file Table1.doc]

**Supplementary Table S1**. Collinearity analysis

|  | **VIF** | | | | | | | |
| --- | --- | --- | --- | --- | --- | --- | --- | --- |
|  | **LCI** | **AIP** | **Non-HDL-C** | **AC** | **CRIⅠ** | **CRIⅡ** | **RC** | **RC/HDL-C** |
| Age | 1.3 | 1.3 | 1.3 | 1.3 | 1.3 | 1.3 | 1.3 | 1.3 |
| Gender | 1.8 | 1.8 | 1.8 | 1.8 | 1.8 | 1.8 | 1.8 | 1.8 |
| BMI | 1.3 | 1.3 | 1.2 | 1.3 | 1.3 | 1.2 | 1.2 | 1.3 |
| SBP | 1.9 | 1.9 | 1.9 | 1.9 | 1.9 | 1.9 | 1.9 | 1.9 |
| DBP | 1.8 | 1.8 | 1.8 | 1.8 | 1.8 | 1.8 | 1.8 | 1.8 |
| FPG | 1.1 | 1.1 | 1.1 | 1.1 | 1.1 | 1.1 | 1.1 | 1.1 |
| ALT | 3.2 | 3.2 | 3.2 | 3.2 | 3.2 | 3.2 | 3.2 | 3.2 |
| AST | 2.9 | 2.9 | 2.9 | 2.9 | 2.9 | 2.9 | 2.9 | 2.9 |
| BUN | 1.1 | 1.2 | 1.1 | 1.1 | 1.1 | 1.1 | 1.1 | 1.1 |
| Scr | 1.8 | 1.8 | 1.8 | 1.8 | 1.8 | 1.8 | 1.8 | 1.8 |
| Smoking status | 1.1 | 1.1 | 1.1 | 1.1 | 1.1 | 1.1 | 1.1 | 1.1 |
| Drinking status | 1.1 | 1.1 | 1.1 | 1.1 | 1.1 | 1.1 | 1.1 | 1.1 |
| Family history of diabetes | 1 | 1 | 1 | 1 | 1 | 1 | 1 | 1 |

Note: The variables with VIF>5 will be regarded as collinear variables and cannot be included in the multiple regression model.

Abbreviations: BMI body mass index, SBP systolic blood pressure, DBP diastolic blood pressure, FPG fasting plasma glucose, ALT alanine aminotransferase, AST aspartate aminotransferase, Scr serum creatinine, BUN blood urea nitrogen, VIF variance inflation factor.

**Supplementary Table S2. The result of two-piecewise logistic regression model**

| **Variables** | **Turning point (K)** | **<K segment effect 1** | **>K segment effect 2** | **The difference between the effect of 2 and 1** | **P for log-likelihood ratio test** |
| --- | --- | --- | --- | --- | --- |
| LCI | 30.997 | 0.995 (0.990, 0.999) ,  P=0.021 | 1.005 (1.000, 1.010) ,  P=0.044 | 1.010 (1.002, 1.019) ,  P=0.012 | 0.012 |
| AIP | -0.375 | 3.034 (1.111, 8.286)  P=0.030, | 0.778 (0.667, 0.906)  P=0.001, | 0.256 (0.089, 0.737)  P=0.012, | <0.001 |
| Non-HDL-C | 2.4 | 0.627 (0.394, 0.996) ,  P=0.048 | 1.027 (0.979, 1.076),  P= 0.277 | 1.638 (1.016, 2.640) ,  P=0.043 | 0.042 |
| AC | 2.616 | 0.820 (0.729, 0.922) ,  P<0.001 | 1.042 (0.982, 1.107),  P= 0.175 | 1.271 (1.090, 1.482) ,  P=0.002 | 0.002 |
| CRI-I | 3.616 | 0.820 (0.729, 0.922) ,  P<0.001 | 1.042 (0.982, 1.107) ,  P=0.175 | 1.271 (1.090, 1.482) ,  P=0.002 | 0.002 |
| CRI-II | 2.504 | 0.872 (0.785, 0.968) P=0.010 | 1.190 (1.057, 1.341)  P=0.004 | 1.366 (1.128, 1.653)  P=0.001 | 0.001 |
| RC | 0.15 | 0.075 (0.025, 0.228) ,  P<0.001 | 0.942 (0.857, 1.034),  P=0.207 | 12.551 (4.031, 39.076),  P<0.001 | <0.001 |
| RC/HDL-C ratio | 0.106 | 0.014 (0.003, 0.073) ,  P<0.0001 | 0.926 (0.840, 1.020),  P=0.119 | 66.327 (12.425, 354.072) ,  P<0.001 | <0.001 |
| TC | 3.64 | 0.656 (0.426, 1.011)，P=0.06 | 1.045 (0.999, 1.092), P=0.05 | 1.592 (1.020, 2.486), P<0.05 | 0.041 |
| TG | 0.59 | 5.248 (1.561, 7.639)，P=0.007 | 0.963 (0.927, 1.000), P<0.05 | 0.183 (0.054, 0.620), P=0.006 | 0.007 |
| HDL-C | 1.17 | 0.811 (0.518, 1.272), P=0.362 | 1.341 (1.130, 1.591) ,  P<0.001 | 1.652 (0.961, 2.841), P=0.069 | 0.070 |
| LDL-C | 1.92 | 0.456 (0.273, 0.760), P=0.003 | 1.103 (1.041, 1.170), P=0.001 | 2.421 (1.424, 4.115), P=0.001 | 0.001 |

Abbreviations: TC total cholesterol, TG triglyceride, HDL-C high-density lipoprotein cholesterol, LDL-C low-density lipoprotein cholesterol, LCI lipoprotein combine index, AIP atherogenic index of plasma, AC atherogenic coefficient, CRI-I Castelli’s index-I, CRI-II Castelli’s index-II, RC remnant cholesterol.

**Supplementary Table S3. Association of Non-Traditional Lipid Parameters with Reversion to Normoglycemia in Different Models: Excluding Patients with Baseline Dyslipidemia**

|  | **Model 1** |  | **Model 2** |  | **Model 3** |
| --- | --- | --- | --- | --- | --- |
|  | **OR (95%CI)** |  | **OR (95%CI)** |  | **OR (95%CI)** |
| **LCI** | 0.964 (0.957, 0.970) |  | 0.987 (0.979, 0.995) |  | 0.990 (0.982, 0.998) |
| **AIP** | 0.291 (0.237, 0.357) |  | 0.628 (0.495, 0.797) |  | 0.708 (0.551, 0.910) |
| **Non-HDL-C** | 0.763 (0.688, 0.846) |  | 0.983 (0.879, 1.098) |  | 0.974 (0.868, 1.094) |
| **AC** | 0.700 (0.638, 0.768) |  | 0.935 (0.842, 1.039) |  | 0.936 (0.839, 1.045) |
| **CRI-I** | 0.700 (0.638, 0.768) |  | 0.935 (0.842, 1.039) |  | 0.936 (0.839, 1.045) |
| **CRI-II** | 0.694 (0.613, 0.787) |  | 0.979 (0.851, 1.125) |  | 0.983 (0.850, 1.137) |
| **RC** | 0.576 (0.489, 0.679) |  | 0.822 (0.689, 0.980) |  | 0.813 (0.677, 0.976) |
| **RC/HDL-C ratio** | 0.505 (0.416, 0.613) |  | 0.805 (0.652, 0.994) |  | 0.800 (0.643, 0.995) |
| TC | 0.900 (0.814, 0.995) |  | 1.028 (0.923, 1.145) |  | 1.016 (0.908, 1.137) |
| TG | 0.652 (0.605, 0.703) |  | 0.833 (0.767, 0.904) |  | 0.868 (0.796, 0.946) |
| HDL-c | 2.206 (1.753, 2.776) |  | 1.315 (1.006, 1.718) |  | 1.280 (0.970, 1.689) |
| LDL | 0.931 (0.824, 1.052) |  | 1.087 (0.955, 1.238) |  | 1.081 (0.945, 1.237) |

Model 1: we did not adjust covariates

Model 2: we adjust gender, age, BMI, SBP, DBP, family history of diabetes, smoking and drinking status

Model 3: further adjusted for FPG, ALT, AST, CCR, and BUN based on model 2

**Supplementary Table S4. Diagnostic Performance of Lipid Parameters for Identifying Normoglycemia: AUC, Optimal Threshold, Sensitivity, and Specificity—Analysis Excluding Patients with Baseline Dyslipidemia**

| **Variables** | **AUC** | **95%CI low** | **95%CI up** | **Best threshold** | **Specificity** | **Sensitivity** |
| --- | --- | --- | --- | --- | --- | --- |
| AIP | 0.5878 | 0.5739 | 0.6017 | -0.1362 | 0.6618 | 0.4760 |
| LCI | 0.5796 | 0.5656 | 0.5936 | 8.0489 | 0.6585 | 0.4642 |
| non-HDL-C | 0.5361 | 0.5220 | 0.5503 | 3.2400 | 0.4201 | 0.6330 |
| AC | 0.5590 | 0.5449 | 0.5731 | 2.0221 | 0.7298 | 0.3744 |
| CRI-Ⅰ | 0.5590 | 0.5449 | 0.5731 | 3.0221 | 0.7298 | 0.3744 |
| CRI-Ⅱ | 0.5448 | 0.5307 | 0.5590 | 1.9311 | 0.4575 | 0.6233 |
| RC | 0.5509 | 0.5367 | 0.5651 | 0.3600 | 0.7409 | 0.3468 |
| RC/HDL-C | 0.5564 | 0.5423 | 0.5706 | 0.2770 | 0.6986 | 0.3935 |
| TC | 0.5141 | 0.4998 | 0.5283 | 4.8950 | 0.2373 | 0.7925 |
| TG | 0.5843 | 0.5703 | 0.5983 | 1.0750 | 0.6241 | 0.5180 |
| HDL-c | 0.5504 | 0.5362 | 0.5645 | 1.3750 | 0.5631 | 0.5187 |
| LDL | 0.5054 | 0.4912 | 0.5196 | 2.1050 | 0.8705 | 0.1477 |

**Supplementary Table S5. Subgroup analysis of Associations Between AIP and reversion to normoglycemia**

|  | Number | OR (95% CI) | | |  | P for interaction |
| --- | --- | --- | --- | --- | --- | --- |
|  |  | Q1 | Q2 | Q3 | Q4 |  |
| Sex |  |  |  |  |  | 0.76 |
| Female | 5260 | Ref | 0.99 (0.85, 1.15) | 0.88 (0.74, 1.05) | 0.84 (0.69, 1.01) |  |
| Male | 9475 | Ref | 1.01 (0.88, 1.16) | 0.82 (0.71, 0.94) | 0.87 (0.76, 1.01) |  |
| Age |  |  |  |  |  | 0.43 |
| < 50 | 8630 | Ref | 0.92 (0.80, 1.06) | 0.83 (0.72, 0.97) | 0.78 (0.67, 0.91) |  |
| ≥ 50 | 6105 | Ref | 1.03 (0.89, 1.20) | 0.80 (0.69, 0.93) | 0.90 (0.77, 1.05) |  |
| BMI |  |  |  |  |  | 0.36 |
| ≥ 24 | 8630 | Ref | 1.16 (1.00, 1.35) | 0.91 (0.78, 1.05) | 0.93 (0.80, 1.08) |  |
| < 24 | 6105 | Ref | 0.85 (0.74, 0.97) | 0.77 (0.66, 0.91) | 0.80 (0.67, 0.96) |  |
| family histroy of diabetes | | |  |  |  | 0.95 |
| No | 14353 |  | 1.01 (0.91, 1.12) | 0.85 (0.76, 0.95) | 0.87 (0.78, 0.97) |  |
| Yes | 382 |  | 0.66 (0.34, 1.29) | 0.49 (0.25, 1.00) | 0.79 (0.39, 1.57) |  |

Models adjusted for the same covariates as in model 3
